# MCU-knockdown attenuates high glucose-induced inflammation through regulating MAPKs/NF-κB pathways and ROS production in HepG2 cells

**DOI:** 10.1371/journal.pone.0196580

**Published:** 2018-04-30

**Authors:** Ghodratollah Panahi, Parvin Pasalar, Mina Zare, Rosario Rizzuto, Reza Meshkani

**Affiliations:** 1 Department of Biochemistry, Faculty of Medicine, Tehran University of Medical Sciences, Tehran, I.R Iran; 2 Recombinant Protein Laboratory, Department of Biochemistry, Shiraz University of Medical Sciences, Shiraz, Iran; 3 Department of Biomedical Sciences, University of Padova, Padua, Italy; Tohoku University, JAPAN

## Abstract

Mitochondrial Ca^2+^ is a key regulator of organelle physiology and the excessive increase in mitochondrial calcium is associated with the oxidative stress. In the present study, we investigated the molecular mechanisms linking mitochondrial calcium to inflammatory and coagulative responses in hepatocytes exposed to high glucose (HG) (33mM glucose). Treatment of HepG2 cells with HG for 24 h induced insulin resistance, as demonstrated by an impairment of insulin-stimulated Akt phosphorylation. HepG2 treatment with HG led to an increase in mitochondrial Ca^2+^ uptake, while cytosolic calcium remained unchanged. Inhibition of MCU by lentiviral-mediated shRNA prevented mitochondrial calcium uptake and downregulated the inflammatory (TNF-α, IL-6) and coagulative (PAI-1 and FGA) mRNA expression in HepG2 cells exposed to HG. The protection from HG-induced inflammation by MCU inhibition was accompanied by a decrease in the generation of reactive oxygen species (ROS). Importantly, MCU inhibition in HepG2 cells abrogated the phosphorylation of p38, JNK and IKKα/IKKβ in HG treated cells. Taken together, these data suggest that MCU inhibition may represent a promising therapy for prevention of deleterious effects of obesity and metabolic diseases.

## Introduction

The evidence suggests that insulin resistance in skeletal muscle, adipose and liver tissues plays an important role in development and initiation of type 2 diabetes (T2D) [1]. In recent years, there has been strong evidence that hyperglycemia, hyperinsulinemia, hyperlipidemia, and pro-inflammatory states contribute to development of insulin resistance [2]. The liver as a vital member of the vertebrates plays a central role in coordination of the entire metabolism. Some of the main functions of the liver include gluconeogenesis, glycogenolysis, glycogenesis, lipogenesis, cholesterol synthesis, synthesis of blood coagulation factors such as fibrinogen and plasminogen activator inhibitor-1 (PAI-1). Liver insulin resistance leads to increased production of glucose, lipoproteins, inflammatory factors such as TNF-α and interleukin 6 (IL-6), and coagulation factors such as fibrinogen A (FGA), fibrinogen B (FGB) and plasminogen activator inhibitor-1 (PAI). The consequence of these disorders is to exacerbate insulin resistance in the liver, as well as in peripheral tissues [3]. Hyperglycemia is considered as one of the most important causes of insulin resistance in liver cells. Glucose toxicity has been proposed to induce insulin resistance by different mechanisms including the formation of advanced glycosylation end products (AGEs), glucosamine production, increased protein kinase C activity, glucose auto-oxidation and a moderate increase in the level of glycolytic reactions [4]. All of these processes are usually involved in the formation of reactive oxygen species (ROS) [5]. On the other hand, inflammation is one of the most important factors in the pathogenesis of hepatic insulin resistance. A number of epidemiological and animal studies have examined the relationship between hepatic insulin resistance and increased production of inflammatory factors such as TNF-α and IL-6 [6, 7].

One critical function of the liver is the integration of multiple signals to maintain normal blood glucose levels. Many of the functions of the liver including glucose metabolism, biliary secretion, mitochondrial physiology, and cell regeneration are regulated by Ca^+2^ [8]. Cytoplasmic calcium fluctuations play a crucial role in regulating these signals. One of the mechanisms controlling the cytoplasmic calcium fluctuations is the absorption of calcium by mitochondria [9]. Ca^2+^ uptake into mitochondria is mediated by the “mitochondrial calcium uniporter,” (MCU) a multisubunit Ca^2+^ channel complex [10]. Mitochondrial Ca^2+^ regulates several basic biological functions, including the efficiency of mitochondrial ATP production and the onset of mitochondria-mediated cell death [10]. The excessive increase in mitochondrial calcium is also associated with the pathological conditions such as apoptosis or necrosis [11]. It was reported that increase in mitochondrial Ca^+ 2^ is associated with the accumulation of ROS, leading to the stable opening of the permeability transfer pores sensitive to cyclosporine (PTP), causing a rapid decomposition of ΔΨm and mitochondrial swelling, loss of pyridine nucleotides and cytochrome c, ATP drainage and ultimately cell death [12]. Given the crucial role of mitochondrial calcium in generation of ROS and because high glucose, known as inducer of pro-inflammatory responses, has been implicated in oxidative stress conditions, we in the present study questioned whether mitochondrial calcium is involved in high glucose (HG)-induced inflammatory and coagulative responses in HepG2 cells. In this study we evaluated the effect of mitochondrial calcium inhibition on the expression of pro-inflammatory and coagulation factors in HepG2 cells exposed to high concentration of glucose.

## Methods & material

### Materials

Dulbecco’s modified Eagle’s medium (DMEM), fetal bovine serum (FBS), and trypsin EDTA were purchased from Gibco (Gibco, Germany). Tissue culture flasks and disposable plastic ware purchased from Greiner Bio-One (Frickenhausen, Germany). N-Acetyl-L-cysteine and secondary antibodies were purchased from Sigma Aldrich (Taufkirchen, Germany). All primary antibodies were purchased from Abcam (Cambridge, UK). Polyvinylidene difluoride (PVDF) membrane was from Millipore (Schwal-bach, Germany). ECL reagents were from Amersham Pharmacia Corp. (Piscataway, NJ, USA). DHE and MitoSOX red were from Invitrogen (San Diego, CA, USA).

### Cell culture and lentivirus infection

HepG2 and HEK T293 cells were purchased from the Iranian Biological Resource Center (IBRC). Cells were cultured at 37°C (in an atmosphere of 5% CO2) in DMEM containing 10% FBS, and 1% penicillin–streptomycin. In order to induce insulin resistance, HepG2 cells were treated with 33 mM d-glucose (HG). d-mannitol (27.5 mM mannitol) was used as an osmotic control in normal glucose (NG) (5.5mM glucose) treated sells. Selection of the time and dose of glucose treatment were based on previously published study [13].

Lentiviruses were prepared as previously described [13]. Briefly, the transfer and packaging (PMD2 and PUMVC) plasmids were transfected into T293 packaging cells. Viral supernatants were collected after 48–72 h transfection and filtered on 0.44 μm filters. HepG2 cells were infected with viral supernatants of SC (scramble) and shRNA-MCU in the presence of 10 μg/ml polybrene for 48 hours.

### Real time PCR

RNA was isolated using GeneAll RibospinTM total RNA purification kit (GeneAll Biotechnology, South Korea). Complementary DNA (cDNA) was reverse transcribed using a RevertAid First Strand cDNA Synthesis Kit (Thermo Fisher Scientific). Gene expression analysis was evaluated by qRT-PCR using SYBR Green RealQ Plus 2x Master Mix Green (Ampliqon) on Corbett Rotor Gene 6000 Light Cycler (Qiagen, Hilden, Germany). The levels of the target gene transcripts were normalized relative to β-actin. The 2^-dCt method was used to calculate the relative expression.

### Western blot analysis

Western-blot analysis was carried out according to our previous report [14]. The antibodies used were MCU, p-JNK, p-ERK, p-P38, JNK, ERK, p38 and Ikkα/β (Cell Signaling Technology, Beverly, MA, USA) and β-actin (Abcam, Cambridge, MA, USA)

### Measurement of intracellular reactive oxygen species (ROS) level

CM-H_2_DCFDA (Life Technologies) was used in the measurement of the ROS production. Briefly, HepG2 cells were washed twice with PBS and then incubated with CM-H_2_DCFDA for 30min, rinsed, and fluorescence measured. MitoSOX red indicator (Life Technologies) was used to measure mitochondrial superoxide. Briefly, cells were incubated in MitoSOX for 15min, rinsed, and fluorescence time course measured. Fluorescence was measured using both a Carl Zeiss Axiovert 200M Inverted Microscope and an Envision Multilabel Plate Reader (Perkin Elmer, Milan, Italy).

### Calcium measurements

HepG2 cells on a 96 well plate were infected with the adenoviruses expressing the cytosolic (Ad‐cytAEQ) or the low-affinity mitochondrial probe (Ad‐mtAEQmut) [15]. Ad-GFP was used as control for adenoviral transduction. The plates were incubated with 5μM coelenterazine for 1–2 h in Krebs-Ringer modified buffer supplemented with 1mM CaCl2. All aequorin measurements were carried out in krebs-ringer bicarbonate buffer. 100nM bradykinin was used to stimulate Ca^2+^ release. All the experiments were terminated by cell lysis with 100 μM digitonin in a hypotonic Ca^2+^-rich solution (10 mM CaCl_2_ in H_2_O) to discharge the remaining reconstituted active aequorin pool. Signals were measured using an Envision Multilabel Plate Reader (Perkin Elmer, Milan, Italy).

### Statistical analyses

All statistical analyses were conducted using SPSS22. (SPSS, Chicago, IL, USA). Statistical significance was considered at p<0.05. Comparisons among all groups were performed with the one-way analysis of variance with Tukey’s HSD post-test when appropriate. Results are expressed as the mean±SEM of at least three independent experiments.

## Results

**Fig 1:**
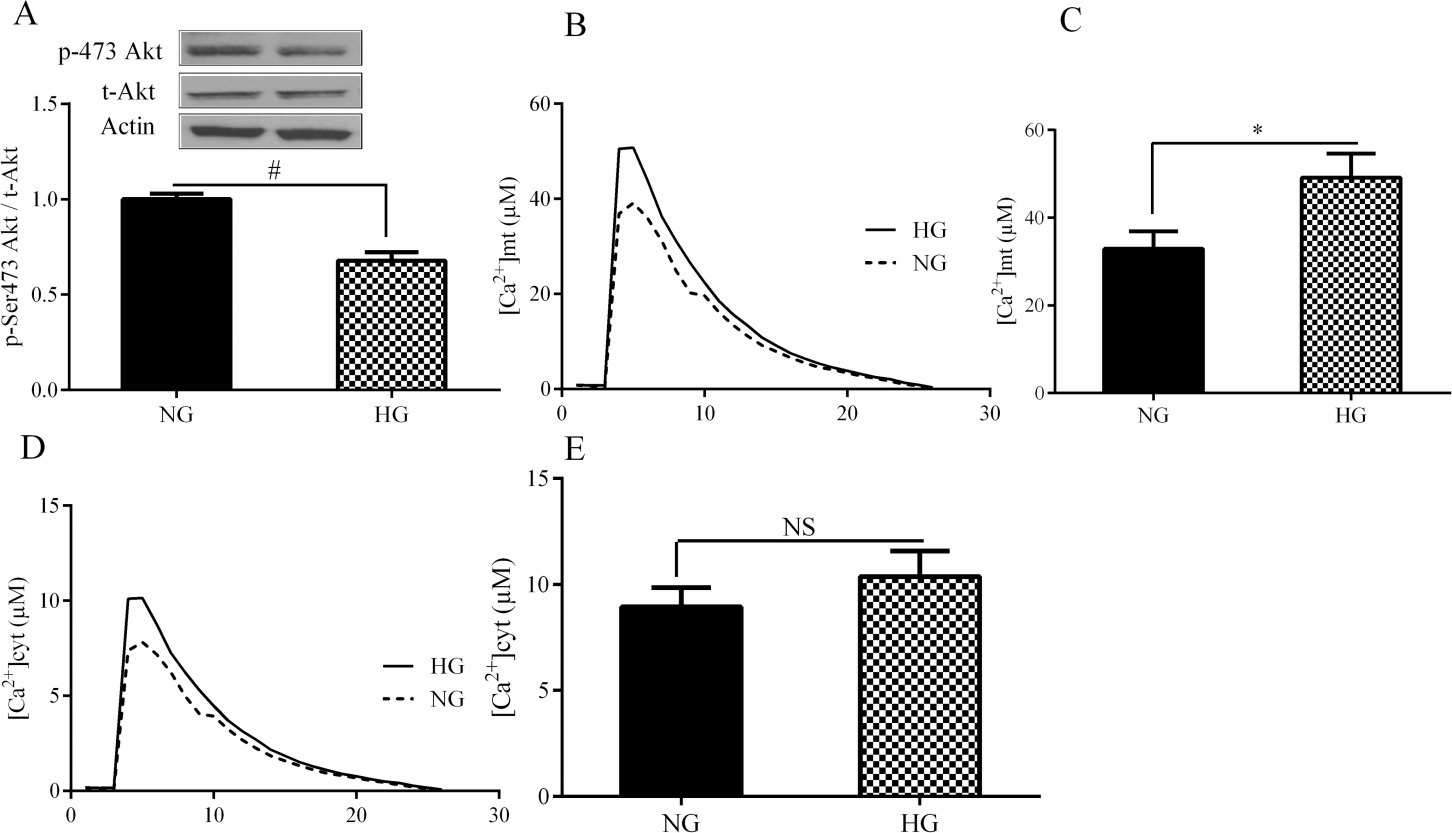
The effect of high glucose on insulin resistance and mitochondrial calcium homeostasis. A: The effect of 33mM glucose (HG) on Akt phosphorylation was performed by western blotting method. B&C: The effect of HG on mitochondrial calcium was assessed using the adenoviruses expressing the low-affinity mitochondrial probe. D&G: The effect of HG on cytosolic calcium was evaluated by adenoviruses expressing the cytosolic probe. Data are shown as a mean ± SEM of at least three separate experiment. NG: normal glucose, HG: high glucose, # <0.05, * <0.01, NS = not significance.

Because of the close link between the oxidative stress and mitochondrial calcium uptake [16], we first studied the correlation between hepatic insulin resistance and changes in mitochondrial calcium homeostasis. Insulin-stimulated Akt phosphorylation reduced after 24 h treatment of HepG2 cells with 33mM glucose (HG) indicating the development of hepatic insulin resistance (Fig 1A). HG treatment led to a marked increase in mitochondrial Ca^2+^ uptake (+75%, Fig 1B), while cytosolic Ca^2+^ remained unchanged (Fig 1C), demonstrating an increased mitochondrial calcium uptake in insulin resistance condition.

**Fig 2:**
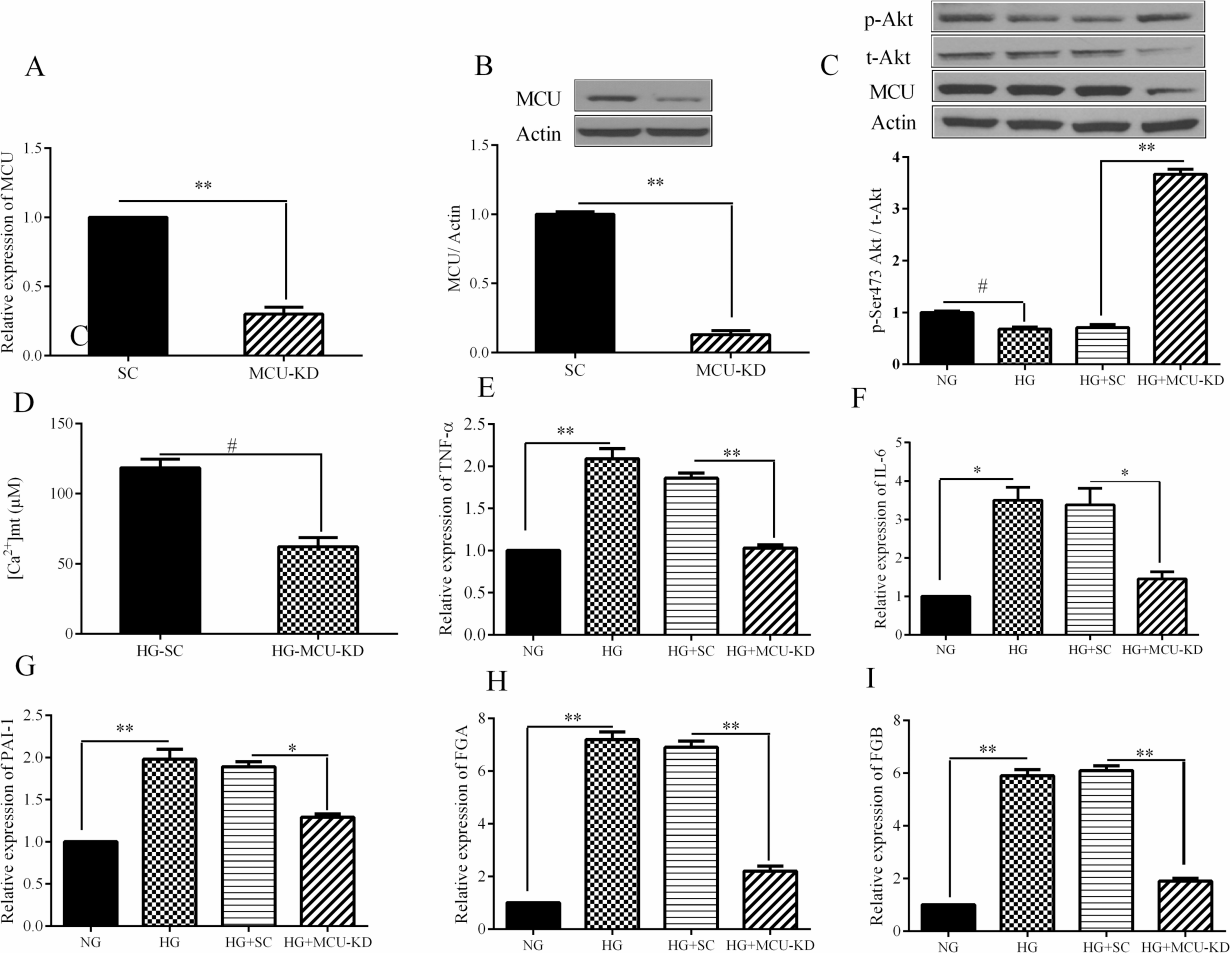
Importance of MCU inhibition in high glucose-induced pro-inflammatory and coagulative responses. HepG2 stable cells were generated by infecting the cells with the supernatants of lentiviruses expressing MCU shRNA. Real-time PCR and western blotting were used to detect MCU mRNA and protein levels in HepG2 stable cells, respectively. A: Protein levels of MCU, B: mRNA level of MCU, C) mitochondrial calcium concentration in MCU-KD cells. D: the effect of MCU inhibition on the mRNA expression of TNF-α (E), IL-6 (F), PAI-1 (G), FGA (H), and FGB(I) were measured using real time PCR. Data are shown as a mean ± SEM of at least three separate experiment. MCU-KD: MCU knockdown cells, SC: Scramble, NG: normal glucose, HG: high glucose, # <0.05, * <0.01, * * <0.001.

Metabolic inflammation is a major player in the development of hepatic insulin resistance [6]. Given the link between high glucose condition and increased mitochondrial calcium in HepG2 cells, we then investigated whether there is an association between MCU and inflammatory cytokines and coagulation factors in hepatocytes. To assess the functional significance of MCU in hepatocytes inflammation, we generated a stable cell line where the expression of MCU was silenced. Lentiviral-mediated shRNA knockdown of MCU in HepG2 resulted in a decrease of mRNA and protein levels of MCU by 70 and 87%, respectively (Fig 2A and 2B). MCU inhibition also reduced HG-induced mitochondrial calcium in HepG2 cells (Fig 2C). Treatment of hepatocytes with HG resulted in a 35% reduction in p-Akt compared to controls, whereas MCU inhibition completely reverted this effect (Fig 2D). We observed that while HG induces the expression of inflammatory and coagulation factors, MCU inhibition resulted in a decrease of TNF-α, IL-6, PAI-1, FGA and FGB expression in HG-treated cells (Fig 2E–2H). These data suggest a strong association between the pro-inflammatory and coagulative responses and mitochondrial Ca^2+^ uptake in liver cells.

**Fig 3:**
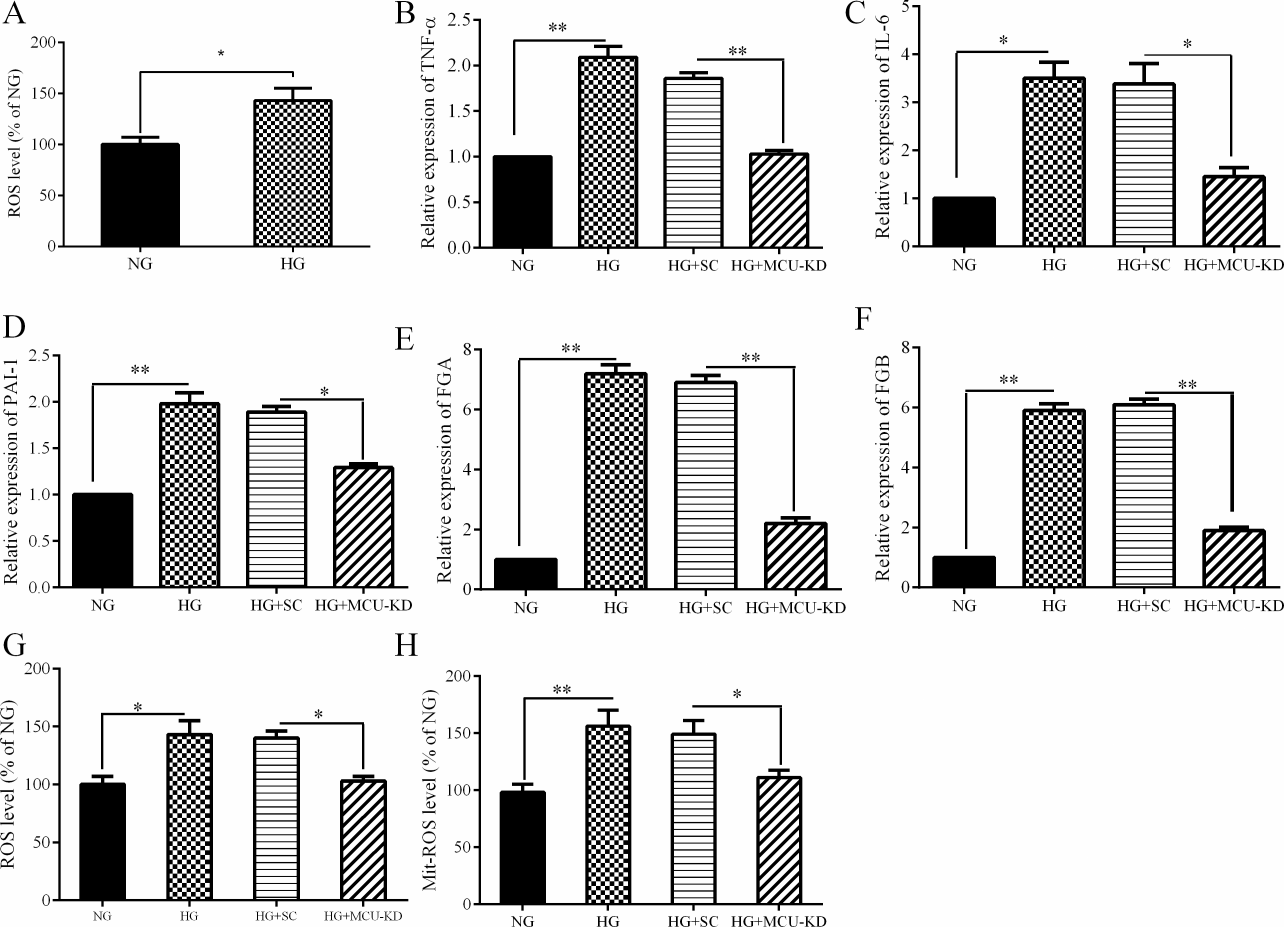
The effect of MCU inhibition on ROS production in HepG2 cells. HepG2 cells were treated with HG for 24 h. A: H_2_O_2_ levels were measured using flow cytometry with DCFH-DA. B: Mitochondrial ROS level using MitoSOX red dye. Data are shown as a mean ± SEM of at least three separate experiment.: MCU knockdown cells, SC: Scramble, NG: normal glucose, HG: high glucose, # <0.05, * <0.01, * * <0.001.

We next sought to identify the mechanism underlying the observed reducing effect of MCU inhibition on hepatic inflammation. Since high glucose is the major cause of oxidative stress, we measured ROS level, the production of which largely relies on the activity of the mitochondrial electron transport complexes. HG treatment results in an increase of both intracellular and mitochondrial ROS levels using DCFDA and MitoSOX dyes, respectively (Fig 3A and 3B). We also observed that MCU knock-down cells exhibited a decrease in ROS level suggesting the key role of MCU in regulating the oxidative stress (Fig 3A and 3B).

**Fig 4:**
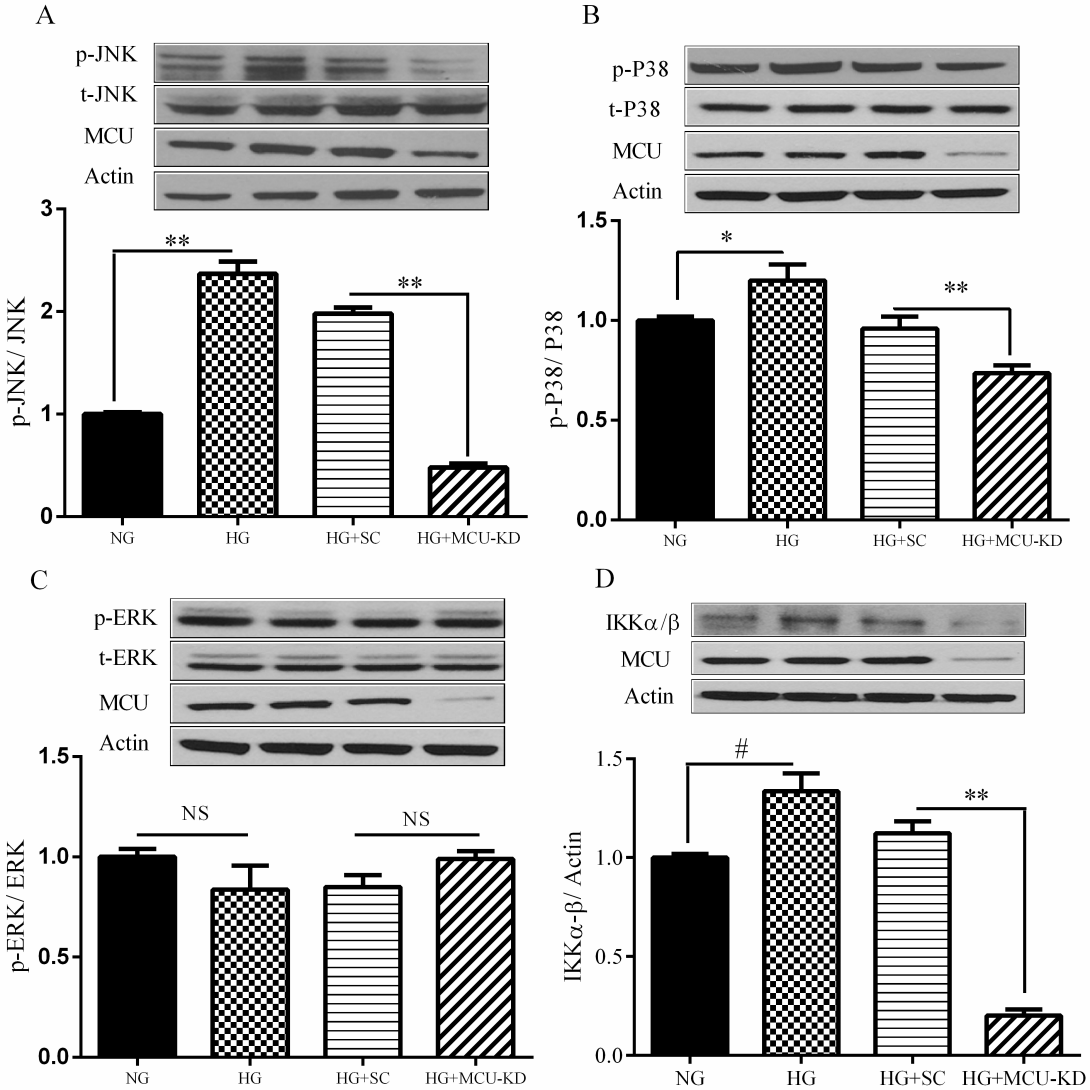
The effect of MCU inhibition on the phosphorylation of MAPK and NF-κB pathway. HepG2 cells were treated with HG for 24 h. After treatment, cells were lysed and protein extracts were immunoblotted with specific antibodies. The effect of MCU inhibition on JNK (A), P38 (B), ERK (C) and IKKα-β (D) phosphorylation has been demonstrated. Data are shown as a mean ± SEM of at least three separate experiment. MCU knockdown cells, SC: Scramble, NG: normal glucose, HG: high glucose, # <0.05, * <0.01, * * <0.001.

Oxidative stress has been shown to induce the inflammatory and coagulative responses by activating the mitogen-activated protein kinases (MAPKs) and nuclear Factor kappa-light-chain-enhancer of activated B cells (NF-kB) pathways [17]. To elucidate the mechanism by which MCU knockdown relieved the increase of inflammation induced by HG, we examined the phosphorylation levels of MAPKs and IKK: IκB kinase (IKKα/IKKβ) in HepG2 cells over HG stimulation. We found that MCU inhibition attenuated HG-induced phosphorylation of c-Jun N-terminal kinase (JNK) and p38, but not extracellulare signal-regulated kinases (ERK) in HepG2 cells (Fig 4A–4C). The level of p-IKKα/IKKβ was also diminished in MCU-knockdown cells treated with HG (Fig 4D). These results suggest that MCU inhibition prevented HG-induced inflammation in HepG2 cells by mechanisms involving the decreasing the activity of the JNK, p38 and NF-κB pathways.

## Discussion

The liver plays a central role in inflammation through its ability to produce acute anti-inflammatory phase proteins and inflammatory cytokines via Kupffer cells and resident lymphocytes [18]. There is evidence that in addition to Kupffer cells and lymphocytes in the liver, hepatocytes can also secrete pro-inflammatory cytokines when they are stimulated by inflammatory markers or fatty acids [19]. The research during the past decades has revealed the mechanism by which hyperglycemia-induced oxidative stress leads to chronic inflammation [20*]*. On the other hand, mitochondrial Ca^2+^ overload has been reported to induce oxidative stress by increasing production of ROS [21]. Therefore, we in the present study aimed to investigate the role of mitochondrial calcium in inflammatory and coagulative responses in hepatocytes by targeting the MCU, a transmembrane protein that allows the passage of calcium ions from the cytosol into mitochondria.

In this study we first investigated the subcellular calcium flux in HG-treated hepatocytes. Chronic HG was associated with a 49% increase in mitochondrial Ca^2+^, while cytosolic Ca^2+^ remained stable. This result is in the line with the data obtained from previous studies. Jorge Suarez et al. showed that calcium transients in cardiomyocytes exposed to HG for 48 h were prolonged compared with transients of the cells cultured in NG [22]. Sand Kim Kumar et al. also observed that HG induces calcium accumulation in the mitochondria of cardiomyocyte H9c2 cells in a time-dependent manner [23]. Mitochondria play significant roles in shaping the Ca^2+^signal released from the endoplasmic reticulum (ER) [24]. Under normal physiological conditions, the bulk of the Ca^2+^ resides within the ER lumen and, during cellular events requiring a Ca^2+^ signal, a small proportion crossing the outer mitochondrial membrane. In pathological conditions such as ER stress, increased release of Ca^2+^ from the ER result in massive and/or a prolonged mitochondrial Ca^2+^ overload [25] In this regard, various pathological conditions including ER stress, oxidative stress, palmitate, and chronic high glucose decreased ER calcium levels in pancreatic β cells [26]. Importantly, the rate of ER calcium-depleted β cells was increased by chronic high glucose [26]. Therefore, because of the stable condition of cytosolic calcium in HG treatment, it appears that increased mitochondrial calcium observed in this study results from increased release of calcium from ER to mitochondria. However, further studies are required to confirm this idea.

In the following experiments, we investigated the relationship between mitochondrial calcium accumulation and the inflammatory and coagulative responses in HepG2 cells by inactivation of MCU. In consistent with the results of the other studies, we found that a 70% decrease of MCU expression was associated with 78% reduction of the absorption of calcium by mitochondria [27]. Insulin-sensitive MCU-knockdown cells exhibited a drastic reduction in the expression of TNF-α, IL-6, PAI-1, FGA and FGB. In agreement with our findings, a recent study reported that a decrease of mitochondrial calcium by Mcub-overexpressing in adipocytes led to a reduction in the release of TNF-α and IL-6 [16]. Furthermore, it was suggested that accumulation of calcium in the mitochondria matrix through the MCU triggers nod-like receptor protein 3 (NLRP3) inflammasome activation and IL-1β release in lung epithelial cells [28]. All together, these data demonstrate a link between mitochondrial calcium and inflammation, thus suggesting a possible role for MCU in the development of obesity-associated metabolic inflammation.

Oxidative stress has been proposed to link with the inflammatory responses. In this regard, it has been demonstrated that ROS can trigger the activation of NF-κB and MAPKs signaling pathways resulting in an increase of the inflammatory processes in various cell types [29, 30]. To investigate the molecular mechanism underlying the anti-inflammatory effect of MCU inhibition, we targeted the oxidative stress and the signaling pathways of MAPKs and NF-kB. In this study HG could promote generation of ROS in HepG2 cells. In support of this finding, it has been demonstrated that oxidative stress was increased following treatment of HepG2 cells with high glucose [31]. MCU inhibition could attenuate HG-induced ROS production in HepG2 cells. Mitochondria are an important source of ROS within most mammalian cells [32]. Given that MCU inhibition completely blocked HG-induced MitoSOX intensity in HepG2 cells, therefore it is plausible to suggest that MCU inactivation may suppress the inflammatory responses by inhibiting mitochondrial ROS production in HepG2 cells. In accordance with this notion, it was reported that decreasing mitochondrial calcium could affect mitochondrial metabolism including the activity of oxidative enzymes, mitochondrial respiration, membrane potential, and ROS production in adipocytes [16]. In the following experiments, we found that HG could induce the phosphorylation of JNK and P38 in HepG2 cells. It is worthy note that a role for JNK and P38 in regulating the inflammatory processes has been previously reported in skeletal muscle and adipose tissues [33, 34]. Interestingly, we were able to demonstrate that MCU inhibition could attenuate HG-induced phosphorylation of JNK and P38. In parallel with the previous reports, we found that HG activates NF-κB pathway as demonstrated by an increased phosphorylation of IKKα/IKKβ [35]. Enhanced phosphorylation of IKKα/IKKβ leads to degradation of IκB protein and subsequent release of NF-κB to translocate to the nucleus [36]. Importantly, we found that MCU inhibition decreased HG-increased phosphorylation of IKKα/IKKβ and thereby NF-κB activity. Collectively, the present data suggest that MCU inhibition ameliorates HG-induced inflammation in HepG2 cells by attenuating oxidative stress and decreasing the activity of JNK, P38 and NF-ΚB pathways. Because of the cancerous nature of the HepG2 cells used in this study, our findings need to be interpreted cautiously and the data should be confirmed in another cell line or primary hepatocytes. However, in support of the data of this study, we should note that HepG2 cells have been widely used as a model of insulin resistance. These cells have ability to synthesize and release bile acids, secrete plasma proteins such as albumin, alpha-fetoprotein, fibrinogen, and apolipoproteins and have receptor for asialoglycoprotein, insulin, transferrin, estrogen, low density lipoprotein (LDL), and high density lipoprotein (HDL) [37–40]. More importantly, a similar profile of the inflammatory responses to different stimuli such as lipopolysaccharide (LPS) and palmitate was reported from HepG2 cells and primary hepatocytes [19, 41].

Akt is responsible primarily for many of the metabolic actions of insulin [42]. Not surprising, therefore, the depression of Akt activation significantly correlated with the increase of insulin resistance condition. In this regard, mice lacking both hepatic isoforms (Akt1:Akt2) show marked glucose intolerance and insulin resistance [43]. Studies have found that chronic and/or increased production of ROS triggers the activation of serine/threonine kinase cascades such as JNK, NF-kB, and others that in turn phosphorylate multiple targets, including the insulin receptor and the insulin receptor substrate (IRS) proteins. Increased serine phosphorylation of IRS and subsequently decreased phosphorylation of Akt explains the molecular basis of oxidative stress-induced insulin resistance [44]. In the present study we observed a lower Akt phosphorylation in HG treated cells. MCU inhibition reversed the effect of HG on Akt phosphorylation indicating an increase in insulin sensitivity of MCU-KD cells. According to above mentioned scenario, our findings of lower mitochondrial calcium and subsequently decrease of oxidative stress, decrease of MAPKs and NF-KB activations may explain the molecular mechanism linking MCU inhibition and increased Akt phosphorylation in MCU-KD cells exposed to chronic HG.

In conclusion, here we demonstrated an association between mitochondrial calcium and the inflammatory and coagulative responses in a model of insulin resistance. Normalizing mitochondrial calcium by inhibiting MCU in HepG2 cells attenuated HG-induced insulin resistance and inflammation by mechanisms involving the inhibition of ROS production and decreasing the activity of the MAPKs and NF-κB signaling pathways. The data suggest that MCU inhibition may represent a promising therapy for prevention of deleterious effects of obesity and metabolic diseases.

## Abbreviations

HG: high glucose
NG: normal glucose
PAI-1: Plasminogen activator inhibitor-1
TNF-α: Tumor Necrosis Factor alpha
IL-6: Interleukin 6
NF-κB: Nuclear Factor kappa-light-chain-enhancer of activated B cells
IKK: IκB kinase
MAPK: Mitogen-Activated Protein Kinases
JNK: c-Jun N-terminal kinase
ERK: Extracellulare signal-Regulated Kinases
ROS: Reactive Oxygen Species
MCU: Mitochondrial Calcium Uniporter
T2D: Type 2 Diabetes
